# Paroxysmal Slow-Wave Events Are Uncommon in Parkinson’s Disease

**DOI:** 10.3390/s23020918

**Published:** 2023-01-13

**Authors:** Dan Z. Milikovsky, Yotam Sharabi, Nir Giladi, Anat Mirelman, Ronen Sosnik, Firas Fahoum, Inbal Maidan

**Affiliations:** 1 Department of Neurology, Neurological Institute, Tel-Aviv Sourasky Medical Center, Tel-Aviv 6423906, Israel; 2 Sackler School of Medicine, Tel-Aviv University, Tel-Aviv 6997801, Israel; 3 Laboratory of Early Markers of Neurodegeneration (LEMON), Neurological Institute, Tel-Aviv Sourasky Medical Center, Tel-Aviv 6423906, Israel; 4 Department of Biomedical Engineering, Engineering Faculty, Tel Aviv University, Tel-Aviv 6997801, Israel; 5 Sagol School of Neuroscience, Tel-Aviv University, Tel-Aviv 6997801, Israel; 6 Faculty of Engineering, Holon Institute of Technology (HIT), Holon 5810201, Israel; 7 Epilepsy and EEG Unit, Neurological Institute, Tel-Aviv Sourasky Medical Center, Tel-Aviv 6423906, Israel

**Keywords:** Parkinson’s disease, EEG, paroxysmal slow-wave events, epilepsy, biomarker

## Abstract

Background: Parkinson’s disease (PD) is currently considered to be a multisystem neurodegenerative disease that involves cognitive alterations. EEG slowing has been associated with cognitive decline in various neurological diseases, such as PD, Alzheimer’s disease (AD), and epilepsy, indicating cortical involvement. A novel method revealed that this EEG slowing is composed of paroxysmal slow-wave events (PSWE) in AD and epilepsy, but in PD it has not been tested yet. Therefore, this study aimed to examine the presence of PSWE in PD as a biomarker for cortical involvement. Methods: 31 PD patients, 28 healthy controls, and 18 juvenile myoclonic epilepsy (JME) patients (served as positive control), underwent four minutes of resting-state EEG. Spectral analyses were performed to identify PSWEs in nine brain regions. Mixed-model analysis was used to compare between groups and brain regions. The correlation between PSWEs and PD duration was examined using Spearman’s test. Results: No significant differences in the number of PSWEs were observed between PD patients and controls (*p* > 0.478) in all brain regions. In contrast, JME patients showed a higher number of PSWEs than healthy controls in specific brain regions (*p* < 0.023). Specifically in the PD group, we found that a higher number of PSWEs correlated with longer disease duration. Conclusions: This study is the first to examine the temporal characteristics of EEG slowing in PD by measuring the occurrence of PSWEs. Our findings indicate that PD patients who are cognitively intact do not have electrographic manifestations of cortical involvement. However, the correlation between PSWEs and disease duration may support future studies of repeated EEG recordings along the disease course to detect early signs of cortical involvement in PD.

## 1. Introduction

Parkinson’s disease (PD) is defined primarily as a motor disorder; however, in recent years PD has been increasingly recognized as a heterogeneous multisystem disorder with a wide variety of non-motor symptoms [1,2]. The progression of PD-related pathology from specific subcortical sites to cortical regions supports this wide involvement of motor and non-motor symptoms [3]. Lesions initially occur in the anterior olfactory nucleus, expand into brain stem areas, and take an upward course involving cortical areas [3]. Therefore, cognitive impairment is a common non-motor symptom in PD and the majority of individuals who survive more than 10 years following PD diagnosis will suffer from dementia, with huge implications for the patients, their families and the healthcare system [4]. However, the presentation and timing of cognitive impairments in PD are variable, making the predictive biomarkers of cognitive decline in PD crucial for both research and clinical practice [4,5,6].

Scalp electroencephalogram (EEG) are an accessible low-cost modality employed regularly in multiple medical centers, such that EEG-based biomarkers might be useful tools. The slowing of EEG activity that includes an increase in spectral power over lower frequency ranges (δ 1–5 Hz and θ 5–8 Hz) and a decrease in higher frequencies (α 8−12 Hz and β 12−20 Hz) has been observed in PD patients with dementia [7], as well as in patients with other types of dementia including Alzheimer’s disease (AD) [8,9]. Moreover, it was found that PD patients with EEG slowing were at higher risk of developing dementia within 2 to 7 years [7,10].

Recently, a new approach has emerged in the study of EEG slowing in other neurological disorders, postulating that cortical slowing is composed of transient paroxysmal slowing of the cortical network [11,12]. These paroxysmal slow-wave events (PSWEs) are transient low-frequency episodes defined by a median power frequency (MPF) below 6 Hz for at least 5 consecutive seconds in humans and an MPF below 5 Hz for 10 s or more in rodents (Supplementary Figure S1) [11,13]. Investigations of EEG recordings from AD and epilepsy patients revealed that network slowing in these pathologies is discrete and composed of PSWEs. Further research found significant potential in the quantification of PSWEs to serve as diagnostic, predictive, and pharmacodynamic biomarkers in AD and epilepsy [11,12,13]. However, this new approach of PSWE quantification to evaluate cortical slowing has not been tested in patients with PD.

Given the EEG slowing observed in PD patients with cognitive decline, we hypothesized that network slowing in PD would be composed of discrete events, as in other neurological diseases, already demonstrating cortical involvement at early stages. Therefore, we implemented a PSWE-detection algorithm [11] over recordings from early-stage PD patients, aged-matched controls, and juvenile myoclonic epilepsy (JME) patients that served as a positive PSWEs control. Our main goal in this study was to compare between healthy controls and PD patients.

## 2. Materials and Methods

### 2.1. Participants

In total, 77 subjects—31 PD patients, 28 healthy older adults, and 18 epilepsy patients who served as a control group to the presence of PSWEs—participated in the study. The participants were recruited from the outpatient clinics of the movement disorders unit and the epilepsy and EEG unit at the Tel Aviv Sourasky Medical Center (TASMC), and from the community. The inclusion criteria for the PD patients were PD diagnosis according to the UK Brain Bank criteria, Hoehn and Yahr between 1 and 2, MOCA > 21, and between 40 to 80 years old. Healthy older adults were included if they were 40 to 80 years old, living in the community or assisted living facility, capable of functioning independently. The inclusion criterion for the epilepsy group was a diagnosis of juvenile myoclonic epilepsy (JME). Participants were excluded if they had: any brain surgery in the past; history of stroke or head injury; a significant central nervous system (CNS) disorder other than PD or epilepsy; suffered from major depressive disorder, schizophrenia, other psychotic disorders, or bipolar disorder. The study was approved by the ethical committee of Tel-Aviv Sourasky Medical Center and was performed according to the principles of the Declaration of Helsinki, approval number 0584-18. All participants gave their informed written consent prior to participation.

### 2.2. Protocol

All the assessments were performed while the patients with PD were in the ON state (about one hour after last medication). Demographic information was obtained for all participants including age, gender, years of education, medications (dose and frequency), and health-related history. Then they performed the Montreal Cognitive Assessment (MOCA) to evaluate cognitive function in different domains (i.e., executive function, visuospatial skills, and memory) [14]. PD severity was evaluated using the MDS-Unified Parkinson’s Disease Rating Scale (UPDRS) [15] and the calculation of levodopa equivalent daily dose (LEDD). After completing the clinical assessment, electrical brain activity was tested using an EEG system (GEODESIC GES 400) with 64 electrodes (60 EEG electrodes and 4 EOG electrodes) at a 250 Hz sampling rate. Electrode position was set according to the International 10-10 standard. Data acquisition was performed over 4 min of rest, with eyes closed. During EEG recordings, participants were instructed to limit eye movement, jaw clenching, or facial expressions that could introduce artifacts into the EEG signal. 

### 2.3. EEG Processing

The data from the EEG recording were processed off-line using MATLAB version 2020a and EEGLAB toolbox version 2020.0. The recorded EEG data were band-pass filtered (1–95 Hz) with a Finite Impulse Response (FIR) filter and band stop filter (45–55 Hz) to eliminate network noises. Channels with prominent artifacts were removed based on visual inspection. Out of 31 PD patients, we excluded 1 electrode from 1 patient. Out of 28 healthy controls, we excluded 1 electrode from 2 patients. Out of 18 epilepsy patients, no electrodes were excluded. Channels’ reference was changed to the average of the 60 EEG channels. Independent component analysis (ICA) was used to detect and eliminate eye movements and muscle artifacts based on visual inspection. The 4 EOG channels were removed from the data prior to further analysis. 

### 2.4. Spatial Averaging

Since we used 60 EEG electrodes, some events were captured in more than one channel. To limit this effect, the channels were averaged spatially into 9 anatomical brain areas, as performed in other studies [10,16] (Supplementary Figure S2). The brain areas were right frontal (area 1); left frontal (area 2); central frontal (area 3); right tempo-parietal (area 4); left tempo-parietal (area 5), central tempo-parietal (area 6); right occipital (area 7); left occipital (area 8); and central occipital (area 9). Signals were not normalized before averaging.

### 2.5. Event Detection and Spectral Analysis

The key feature to identify PSWEs is the Median Power Frequency (MPF). An event was defined as MPF lower than 6 Hz for five consecutive seconds or more, as previously described [11]. Fast Fourier Transform (FFT) was calculated using 2 s window with 1 s overlap between consecutive windows to extract the MPF from each time window. 

### 2.6. Statistical Analyses

The mean and standard deviation of all clinical variables were calculated and evaluated for normality and homogeneity using box plots, scatter plots and Kolmogorov–Smirnov tests. Kruskal–Walls and one-way ANOVA were used to examine differences between groups. Liner mixed-model analysis was used to examine the effects of group (HC, PD, Epilepsy), brain region (frontal, tempo-parietal, occipital), and the interaction between them (group*brain region) on the number of PSWEs. Post hoc analysis with a correction of least significant difference (LSD) for multiple comparisons was performed between the main effects (group and brain region). Correlations between PSWEs, disease duration, and cognitive measures were explored using Spearman correlation analysis.

## 3. Results

As shown in Table 1, no differences in demographics and MOCA score were observed between the PD group and healthy controls. In addition, Table 1 reveals that the patients with PD were at the early stages of the disease based on a relatively short disease duration of 2.3 years in addition to H&Y stage 1–2 as mentioned in the methods. In contrast, the epilepsy group had fewer years of education and scored lower on the MOCA than the healthy control group.

**Figure 1:**
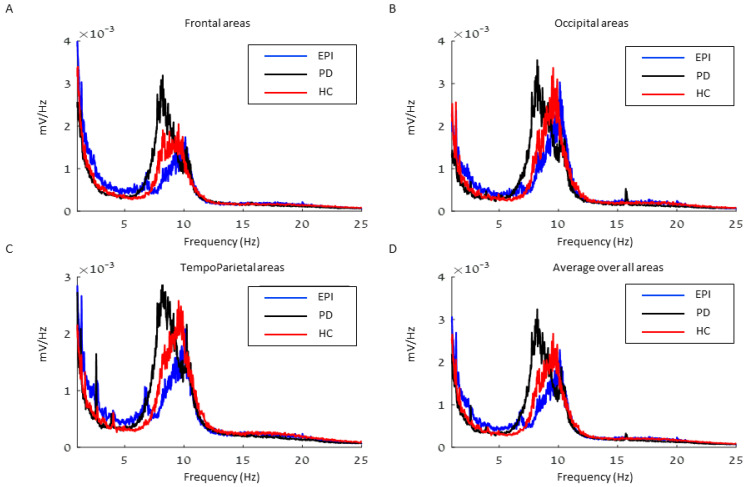
The average spectrum of each group in (**A**) Frontal areas, (**B**) Occipital areas, (**C**) Tempo-Parietal areas, and (**D**) an average over all areas. EPI = Epilepsy, PD = Parkinson’s disease, HC = Healthy control.

**Figure 2:**
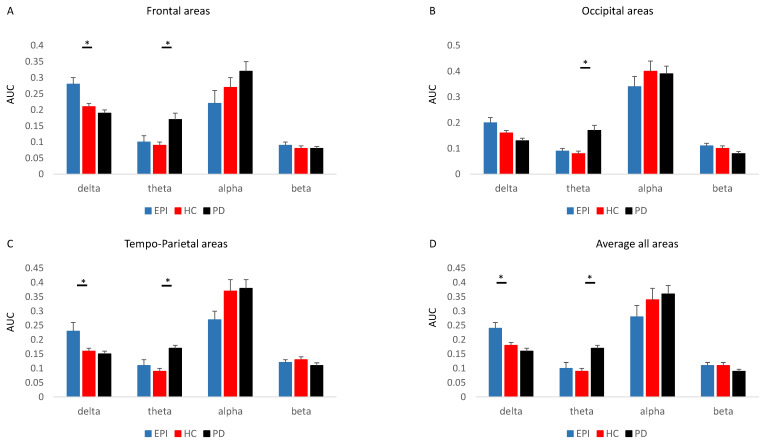
The area under the curve (AUC) of each of the waves for the spectrums in (**A**) Frontal areas, (**B**) Occipital areas, (**C**) Tempo-Parietal areas, and (**D**) Average over all areas. * significant differences.

As shown in Figure 1 and Figure 2, spectral analyses demonstrated significantly higher relative delta power (1 to 5 Hz) in epilepsy patients compared to healthy controls in frontal (*p* = 0.028) and tempo-parietal areas (*p* = 0.046). No significant difference in delta was found in occipital areas (*p* = 0.205). Moreover, no differences in alpha (*p* > 0.100), beta (*p* > 0.592), and theta (*p* > 0.486) bands were observed between healthy controls and epilepsy patients. A comparison between patients with PD and healthy controls revealed significant higher theta relative power (5 to 8 Hz) in patients with PD in all brain areas (frontal: *p* = 0.002, tempo-parietal: *p* = 0.001, occipital: *p* < 0.001). No differences in alpha (*p* > 0.275), beta (*p* > 0.115), and delta (*p* > 0.120) bands were found between patients with PD and healthy controls.

**Figure 3:**
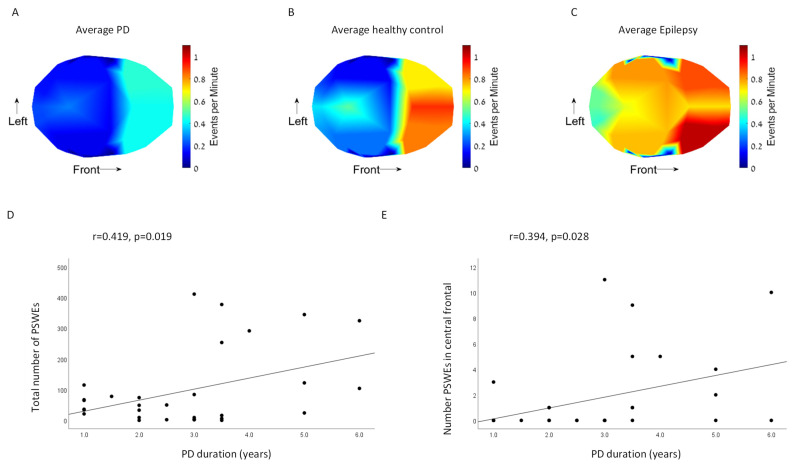
Heatmap of events per minute in (**A**) PD group, (**B**) Healthy control group, and (**C**) Epilepsy control group. Correlations between PD disease duration and number of PSWE in (**D**) all brain areas and (**E**) central frontal area.

Significant differences in the number of PSWEs between the groups were found in specific brain areas (interaction Group x brain area: *p* < 0.001) (Figure 3A–C). Patients with epilepsy showed significantly higher numbers of PSWEs than healthy controls in the right tempo-parietal region (*p* = 0.023) and left tempo-parietal region (*p* < 0.001). To ensure that patients with long continuous PSWEs did not mask the number of PSWEs, we also compared the percentage of time a subject was in a PSWE state. We found similar results, in which epilepsy patients had significantly longer PSWE states compared to healthy controls (*p* = 0.0127). 

In contrast, we did not find a significantly higher rate of PSWEs in PD patients compared with controls (right tempo-parietal: *p* = 0.478, left tempo-parietal: *p* = 0.944). Moreover, PD patients showed a significantly lower number of PSWEs in the central frontal region compared with controls (*p* = 0.041). Healthy controls had a significantly longer percentage of time in a PSWE state compared to PD patients (*p* = 0.018). Comparison between brain areas revealed higher numbers of PSWEs in the frontal areas compared to occipital and tempo-parietal areas in all groups (Brain area effect: *p* < 0.001). 

Patients with PD with longer disease duration demonstrated higher numbers of PSWEs in all brain areas (r = 0.419, *p* = 0.019) (Figure 3D) and in the central frontal area (r = 0.394, *p* = 0.028) (Figure 3E). The number of PSWEs in the central tempo-parietal and occipital areas did not correlate with disease duration (r < 0.268, *p* > 0.145). No correlations were found between the number of PSWEs and MOCA scores.

The definition of PSWEs in humans (a decrease in MPF to below 6 Hz for more than 5 consecutive seconds) was made using analysis of EEG recordings from AD patients and control subjects [11]. These thresholds were found to be valid also in epilepsy patients [11,13]. In this study, we tested, for the first time, EEG recordings of PD patients. Therefore, we performed the analysis using several thresholds (2–8 Hz) as previously described [11]; however, we did not find another threshold that yielded a better separation between PD patients and controls (2 Hz threshold *p* = 0.361, 3 Hz threshold *p* = 0.598, 4 Hz threshold *p* = 0.323, 5 Hz threshold *p* = 0.103, 7 Hz threshold *p* = 0.431 and 8 Hz threshold *p* = 0.944). 

## 4. Discussion

In this study, we evaluated the temporal characteristics of EEG slowing in early-stage PD patients and specifically tested for the presence of PSWEs in this group. We found that EEG recordings from early PD patients show higher θ relative power compared with healthy controls in all brain regions. As a positive control for the presence of PSWEs in short EEG recordings, we used a group of JME patients who demonstrated significantly higher δ relative power composed of PSWEs in bilateral tempo-parietal regions compared with healthy controls. Unlike our hypothesis, PD patients did not show significantly increased PSWEs occurrence compared with controls; however, PSWEs were significantly correlated with disease duration in this group.

In line with the literature, our spectral analyses demonstrated significantly higher relative power of δ in JME patients compared to healthy controls, despite their younger age [17]. Our findings further show higher relative power in δ compared with PD patients (Figure 1 and Figure 2). On the other hand, PD patients presented a higher power of θ band in all brain regions, compared with age-matched controls and JME patients. Increased θ power has been widely reported in PD patients, specifically in PD patients with cognitive decline [10,18]. In our study, the PD patients were at early stages of the disease (H&Y 1–2) and without a cognitive decline. This finding of high θ power already at early stages of PD stresses the potential of EEG as an early predictive biomarker of dementia in PD, thus a long-term follow-up of these patients is necessary.

When searching for the presence of PSWEs, consistent with previous studies we found that epilepsy patients have significantly more PSWEs compared with healthy controls (Figure 3) [11,13]. Unlike our hypothesis, the temporal characteristics of EEG slowing in PD patients did not show a significant difference in the number of PSWEs compared with control (Figure 3). Recent human and animal studies of AD and epilepsy showed that PSWEs are the electrocorticographic manifestations of the blood–brain barrier dysfunction (BBBd) in these pathologies [11,12]. A large body of evidence shows a crucial role for BBBd in the pathogenesis of AD in cortical regions [19,20]. Although BBBd was described in PD, it was not shown in cortical regions [20]. The lack of PSWE prevalence among the PD patients indicates minimal cortical involvement and the absence of cortical BBBd in the early stages of the disease.

Nevertheless, we found that, among PD patients, a higher number of PSWEs correlated with longer disease duration (Figure 3D,E). That might be a preliminary finding that motivates further experiments over groups of patients in more advanced stages of the disease. Specifically, repeated EEG recordings along the disease course may help to predict cortical involvement by finding trends of increase in PSWEs over time. The high MOCA score and lack of cognitive impairment in the PD group (Table 1), explains why no correlation was found between the number of PSWEs and MOCA. A previous study that showed correlation between PSWEs and mini mental state examination (MMSE) score included participants with mild cognitive impairment (MCI) and AD, which can explain the discrepancy between these findings [11].

An additional aspect of this study is the topographical distribution of PSWEs. Employing a 60-channel EEG system, we were able to explore differences in PSWE occurrence between nine brain regions. Previous studies demonstrated that different frequency ratios between brain regions had the largest effect to distinguish between PD patients with and without dementia [10,18], indicating that alterations in topographical distribution may add important information. While our spectral analyses revealed similar topographical distribution, the frequency of PSWEs differ between brain regions. PSWE frequency was higher in frontal regions compared to tempo-parietal and occipital regions in all three groups, indicating that intervals of lower frequency may be an inherent part of frontal lobe functionality. These findings raise important questions regarding the definition of PSWEs in terms of MPF threshold and duration in different regions. An interesting finding observed in the frontal region was the lower PSWE frequency in the PD group. As mentioned, an ICA algorithm was implied to exclude eye-movement-related noise. However, reduced spontaneous eye movement as a manifestation of hypomimia, a common PD symptom, may contribute to this observation. Additionally, similar trends regarding frontal PSWE in epilepsy patients were found in a previous study [13].

Our study is in line with other recent investigations trying to utilize EEG as a non-invasive potential diagnostic biomarker for PD. In one study, a deep learning model was used to analyze EEG recordings and achieved high accuracy for detecting PD and medication status [21]. Another study proposed an ensemble of deep learning models to predict PD using imaging data and developed a software tool for real-time detection [22,23].

This study has several limitations that should be addressed. The EEG recording lasted four minutes, which is relatively short, compared with previous studies that performed 20–30 min of EEG recording [11,13]. Despite the short duration of the resting task, we were able to detect enough PSWEs to expose significant differences between groups. This raises a question regarding the necessity of long recording that increases the burden on the health care system and patients. In addition, our resting task was with eyes closed, which tends to increase α power and, therefore, may decrease the relative power of δ, the band of interest in this study. Future studies should include both eyes open and eyes closed and a comparison between them. The low-frequency and duration thresholds used to define our PSWEs relied on previous work that mainly included patients with AD and epilepsy. As mentioned, we did not find a better threshold for PD. However, we found a significant difference between PD patients and controls in the relative power of theta band (Figure 2). Future studies should examine the potential of paroxysmal bands (5–8Hz) rather than use an upper threshold only. In this study, we averaged the number of channels to represent specific brain areas. Future studies may perform separate analysis for each channel to add information and extend our knowledge. The PD group included patients at early stages of disease with minimal cognitive changes. Future studies should include PD patients at different stages of the disease to reveal the effects of disease progression and specifically of cognition on PSWE occurrence. Our study did not include a modality that can reveal BBBd. Therefore, our conclusions are limited to the electrical brain changes, defined as PSWE. Future studies should combine modalities, such as DCE-MRI and EEG examinations of PD patients, to elucidate the putative role of BBB dysfunction in functional network changes.

## 5. Conclusions

Overall, our study is the first to examine temporal characteristics of EEG slowing in PD and the first to question cortical involvement at early stages of the disease by measuring the presence of PSWEs. The lack of PSWEs emphasizes minimal cortical involvement at early stages of the disease. Significant correlation between PSWEs and PD duration may support future studies of repeated EEG recordings along the disease course to test the potential of PSWEs to predict or detect cortical involvement in PD.

## Figures and Tables

**Table 1:** Participants’ characteristics.

	Healthy (n = 28)	Epilepsy (n = 18)	PD (n = 31)	Healthy/EPI (*p*-Value)	Healthy/PD (*p*-Value)
Age (years)	60.5 ± 7.4	28.5 ± 5.5	63.3 ± 8.2	<0.001	0.181
Gender (M/F)	13/15	7/11	18/13	0.619	0.376
MOCA	27.4 ± 1.9	25.1 ± 3.3	26.9 ± 2.1	0.005	0.353
Disease duration (years)	-	11.6 ± 0.9	2.8 ± 0.3	-	-
LEDD (mg)	-	-	177.9 ± 44.4	-	-

M = Male, F = Female, MOCA = Montreal cognitive assessment, EPI = Epilepsy, PD = Parkinson’s disease. The statistics represent mean ± SD

## Data Availability

Data will be shared upon any reasonable request.

## Supplementary Materials

The following supporting information can be downloaded at: https://www.mdpi.com/article/10.3390/s23020918/s1, Figure S1: An example for a PSWE recorded from a patient: A. EEG trace. B. Median power frequency of the trace in A. C. Magnification of the segment in a dashed rectangle. PSWE—paroxysmal slow wave event. MPF—median power frequency. Figure S2: The nine anatomical brain areas used for signal averaging: (1) Right Frontal, (2) Left Frontal, (3) Central Frontal, (4) Right Tempo-parietal, (5) Left Tempo-parietal, (6) Central Tempo-parietal, (7) Right Occipital, (8) Left Occipital and (9) Central Occipital.
