# Supplementary material for: Paroxysmal Slow-Wave Events Are Uncommon in Parkinson’s Disease
Source: Sensors (Basel). 2023 Jan 13;23(2):918. doi: 10.3390/s23020918 (PMC9862294; doi:10.3390/s23020918)
Supplement: Supplementary file 1 [file sensors-23-00918-s001.zip › Supplementary Figures.pdf]

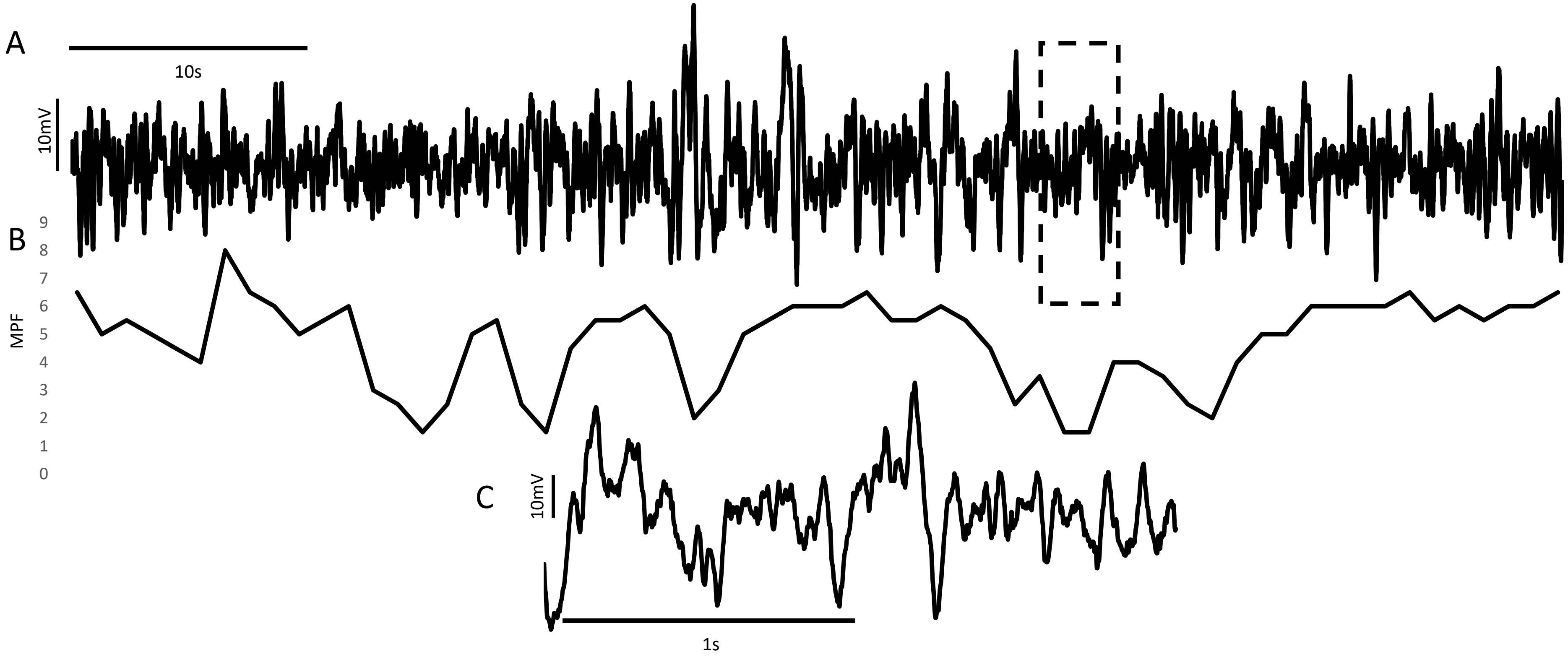


**Supplementary Figure S1:** An example for a PSWE recorded from a patient. A. EEG trace. B. Median power frequency of the trace in A. C. Magnification of the segment in a dashed rectangle. PSWE – paroxysmal slow wave event. MPF – median power frequency.

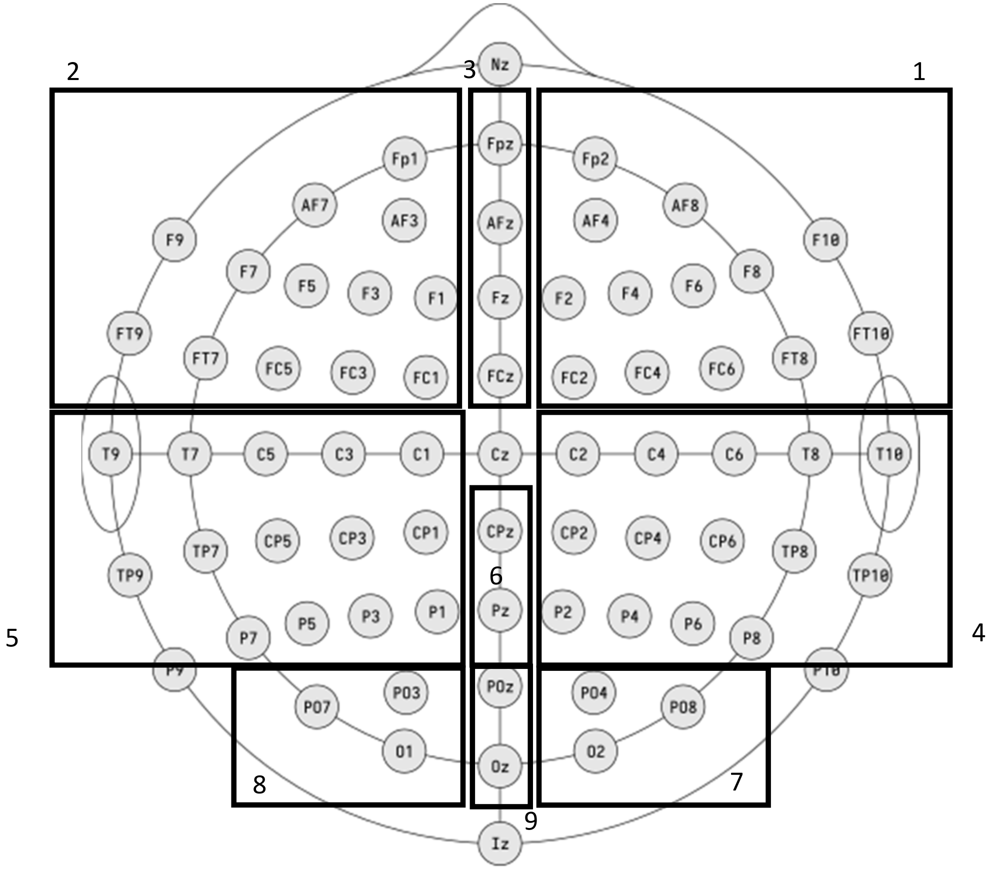


**Supplementary Figure S2:** The nine anatomical brain areas used for signal averaging: (1) Right Frontal, (2) Left Frontal, (3) Central Frontal, (4) Right Tempo-parietal, (5) Left Tempo-parietal, (6) Central Tempo-parietal, (7) Right Occipital, (8) Left Occipital and (9) Central Occipital.
